# Retroperitoneal fibrosis, a rare entity with urorenal and vascular subtypes – preliminary data

**DOI:** 10.1080/0886022X.2022.2064303

**Published:** 2022-04-20

**Authors:** Izabela Łoń, Jacek Lewandowski, Monika Wieliczko, Jolanta Małyszko

**Affiliations:** a Department of Hypertension, Angiology and Internal Diseases, Medical University of Warsaw, Warsaw, Poland; b Department of Nephrology, Dialysis and Internal Diseases, Medical University of Warsaw, Warsaw, Poland

**Keywords:** Retroperitoneal fibrosis, vascular type, urorenal type, acute kidney injury

## Abstract

**Introduction:**

Retroperitoneal fibrosis (RPF) is a rare disease associated with the formation of hard inflammatory and fibrous tissue in the retroperitoneum. Taking into consideration the fact that RPF is a rare disease with different subtypes, we compared the basal clinical and biochemical characteristics of the vascular and urorenal subtypes.

**Patients and methods:**

From January 2005 until December 2021, 27 patients were identified as vascular subtype (18 males) and 11 as urorenal subtype (9 males).

**Results:**

Patients with a primary urorenal origin had significantly worse kidney function as reflected by serum creatinine and eGFR (both *p* < 0.001); they also had higher serum cholesterol (*p* < 0.01). Hypertension, diabetes, hyperlipidemia and nicotinism were significantly more prevalent in vascular subtype (all *p* < 0.001).

**Conclusion:**

Vascular subtype is more prevalent in our study with more cardiovascular risk factor present. Due to the diversity of symptoms, diagnosis of RPF becomes a challenge for specialists as well as therapy.

## Introduction

Retroperitoneal fibrosis (RPF) is a rare disease associated with the formation of hard inflammatory and fibrous tissue in the retroperitoneum [1]. This tissue is generally localized around the aorta below the renal and iliac arteries, usually involving the ureters, less often the vena cava inferior, in the disease process. The etiopathogenesis of this disease still remains an enigma. A local inflammatory response to atherosclerotic plaque antigen, as a starting mechanism of the chronic inflammatory process, is one of the postulated causes. The second is the local autoimmune process.

Taking into account the dominant symptoms and clinical characteristics of patients with RPF we described vascular and urorenal subtypes in the recent review [2]. The vascular subtype includes patients with inflammatory abdominal aortic aneurysm (IAAA) and with RPF around a non-dilated aorta, with numerous risk factors for atherosclerosis. Depending on the width of the aorta surrounded by the inflammatory infiltrate, it can be divided into simple RPF or IAAA. The estimated frequency of IAAA is 2.3% to 10% of all abdominal aneurysms of the aorta [3,4]. A typical vascular subtype patient is an obese or overweight man in the fifth or sixth decade of life, a heavy smoker, with some type of lipid metabolism disorder, and often with hypertension, fasting hyperglycemia, or impaired glucose tolerance. Some patients have concomitant chronic coronary artery syndrome or peripheral atherosclerosis; others have a history of a cardiovascular event, such as a stroke or heart attack. These patients rarely have other autoimmune diseases. Uibu et al. [5] and Goldoni et al. [6] revealed that a history of cigarette smoking and/or exposure to asbestos increases the risk of RPF and a multiplicative effect was found between smoking and both occupation and extraoccupational exposure to asbestos. Clinical features, especially early signs, are nonspecific, which makes diagnosis difficult and often distracting. The most common clinical symptom of both subtypes is pain (in 90% of cases): blunt, regardless of body position, in the lower back, sacro-lumbal, and lateral region, sometimes radiating to the inguinal region and suggesting a renal colic. The pain is persistent, poorly responsive to opiates and often radiates to the testicles [7,8]. One of the most frequent complications of the disease is obstructive uropathy manifesting with hydronephrosis. Involvement of the ureter is uni- or bilateral, often initially asymptomatic and consists of pressing it, usually in the middle part. Significant bilateral ureter stenosis causes acute kidney injury (AKI), and when the ureters are completely closed, the leading symptom is anuria. In one-third of cases, at the moment of diagnosis, the kidney with reduced size is also found, which indirectly indicates a long-term process that initially was unilateral and ureteral obstruction by the fibroinflammatory tissue is the more probable cause of renal damage.

Considering the fact that RPF is a rare disease with different subtypes, we compared the basal clinical and biochemical characteristics of the vascular (cardiology) and urorenal subtypes.

## Patients and methods

The study’s main objective was to evaluate the clinical characteristics of patients with RPF admitted to the Department of Hypertension, Angiology and Internal Medicine and Department of Nephrology, Dialysis and Internal Medicine, Warsaw Medical University. All adult patients that were referred from 2005 with suspected RPF by other specialists, mostly by vascular surgeons to the Department of Hypertension, Angiology and Internal Medicine and by urologists to the Department of Nephrology, Dialysis and Internal Medicine, were included in the study.

We analyzed the medical data from the Clininet system at the hospital to obtain demographic (age, gender, comorbidities etc.) and biochemical data (complete blood count, markers of inflammation, serum creatinine, uric acid, serum lipids, etc.) as well as imaging, endoscopy studies, and all required consults. A positive opinion of our Ethical committee was obtained and informed consent was waived per regulation due to the retrospective design.

## Results

From January 2005 until December 2021, 27 patients were identified as vascular subtype (18 males) and 11 as urorenal subtype (9 males). All data are presented in Table 1. Patients with a primary urorenal origin had significantly worse kidney function as reflected by serum creatinine and eGFR; they also had higher serum cholesterol. Hypertension, diabetes and hyperlipidemia and nicotinism were significantly more prevalent in vascular subtype.

**Table 1.** Clinical and biochemical data of two subtypes of patients with retroperitoneal fibrosis.

	Vascular subtype *n* = 27	urorenal subtype *n* = 11
Age (years)	60.82 ± 9.70	59.09 ± 6.38
Hypertension (%)	74	18***
Diabetes (%)	37	7***
Hyperlipidemia (%)	93	9***
Cigarette smoking (%)	100	9***
Hemoglobin (g/L)	123.4 ± 17.2	119.9 ± 26.0
Leukocyte count (10^9^/L)	8.67 ± 2.38	9.21 ± 3.87
Platelet count (10^9^/L )	268.48 ± 80.09	232.57 ± 78.24
Sodium (mmol/L)	139.77 ± 2.30	139.35 ± 1.57
Potassium (mmol/L)	4.43 ± 0.57	4.70 ± 0.41
CRP (mg/L)	3.7 (1.1–12.9)	17.8 (3.4–45.5)*
Creatinine (µmol/L)	105.19 (86.6–134.37)	330.62 (214.81–707.20)***
Urea (mmol/L)	3.47 ± 2.18	5.14 ± 4.18*
eGFR by CKD-EPI (ml/min/1.72m^2^)	87.95 ± 1.82	6.33 ± 21.98***
Uric acid (µmol/L)	344.98 ± 108.25	376.5 ± 58.88
Cholesterol (mmol/L)	4.55 ± 1.01	5.99 ± 0.51**
HDL (mmol/L)	1.09 ± 0.29	1.55 ± 0.81
LDL (mmol/L)	2.63 ± 0.8	3.09 ± 0.68
Triglycerides (mmol/L)	1.6 ± 0.67	2.26 ± 1.32

Data given as means and SD or median and interquartile ranges.

Conversion factors to SI units are as follows: for creatinine 88.4, for urea 0.357.

*p < 0.05, ***p < 0.001.

**Figure 1.**
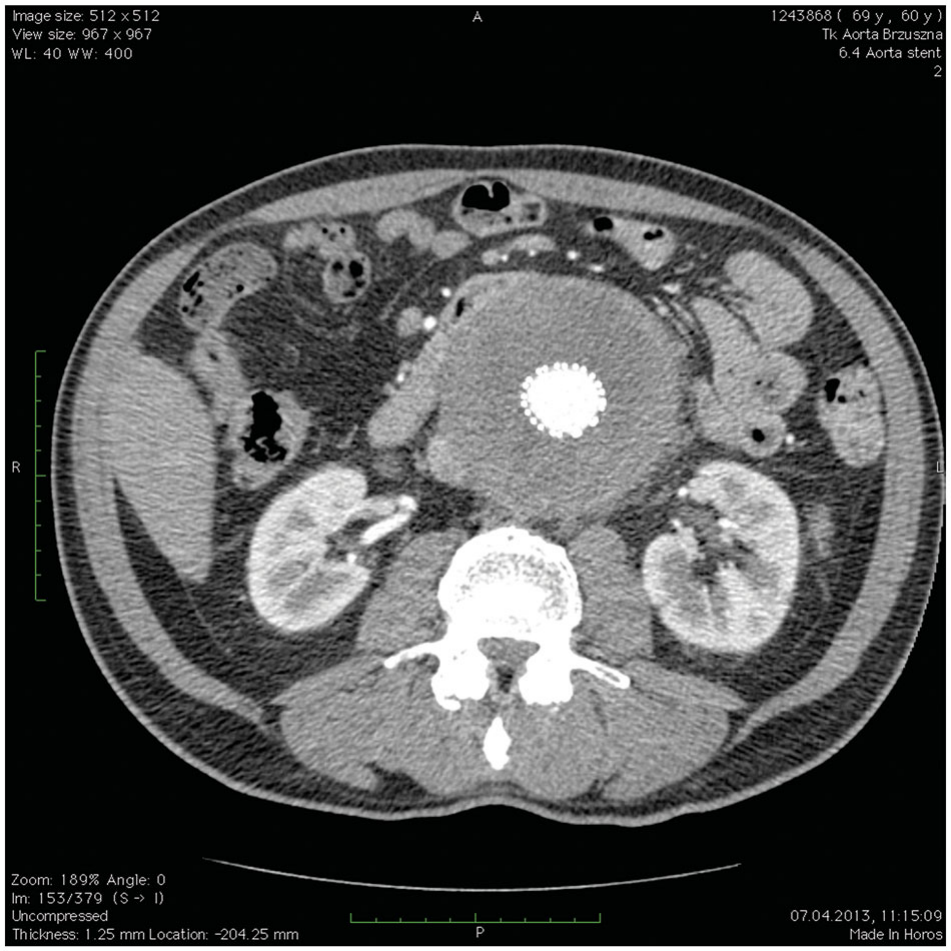
CT scan of subjects with vascular subtype of retroperitoneal fibrosis. Scan shows an aortic inflammatory aneurysm after endovascular stent insertion.

**Figure 2.**
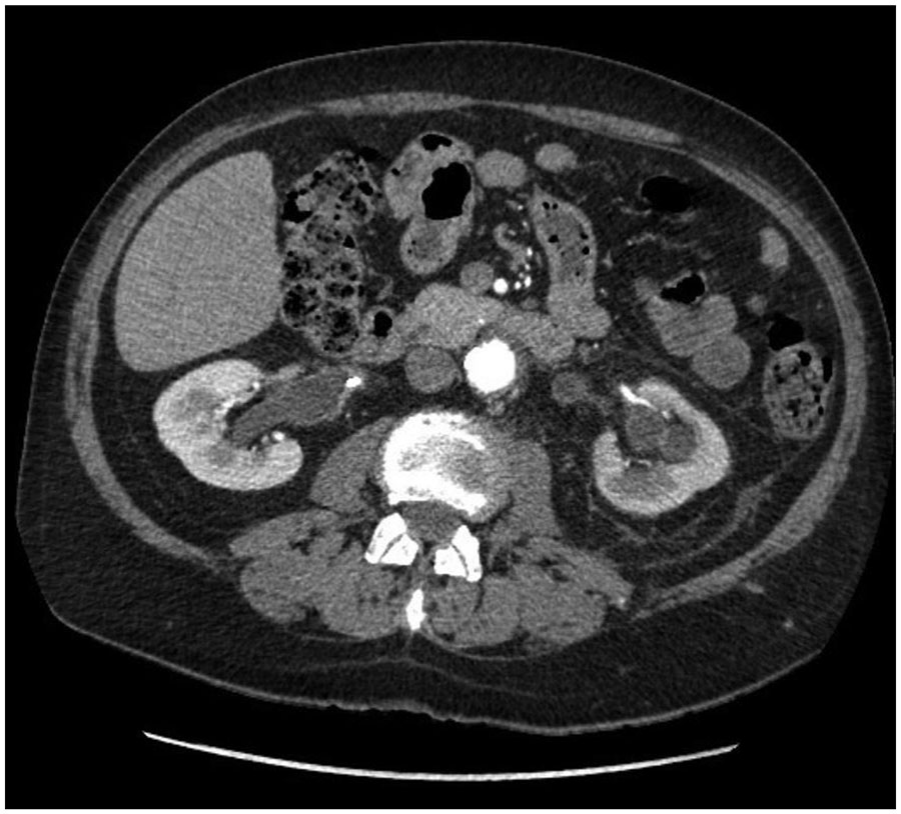
CT scan of subjects with urorenal subtype of retroperitoneal fibrosis. Bilateral hydronephrosis with inflammatory tissue involving ureter.

As there are no radiological, microbiological, or biochemical methods that would uniquely differentiate between primary and secondary fibrosis, at first, we tried to exclude secondary causes, especially malignancies. We performed a series of diagnostic tests, both biochemical and imaging (CT, MRI) or endoscopic, and found that all of the cases were primary RPF. Figure 1 presents vascular and Figure 2 urorenal subtype.

## Discussion

In our clinical practice, we recognized two different subtypes. In the published literature, these subtypes are not discussed. The vascular subtype is a group of patients with IAAA and with RPF around a non-dilated aorta, with numerous risk factors for atherosclerosis. Characteristically, IAAAs are frequently complicated by inflammatory adhesions from retroperitoneal fibrosis involving local structures, increasing the potential for iatrogenic injury in open surgical repair. Recently it has been suggested that idiopathic RPF is seen in the presence of aortic aneurysms as a part of the disease spectrum of chronic periaortitis [9,10]. Although the specific pathogenesis is not well understood but it has been proposed that there is an exaggerated immune response triggered by atherosclerotic plaques in the aorta. Recently, a few case reports on the association between IAAA and RPF were published [11–13]. In the recent study from 2022, Yardimci et al. [14] reported that in the group of 51 patients (37 males), the most common form of chronic periaortitis was idiopathic retroperitoneal fibrosis (82%), followed by inflammatory abdominal aortic aneurysms (12%) and peri-aneurysmal retroperitoneal fibrosis (8%). Some patients diagnosed with idiopathic retroperitoneal fibrosis could be reclassified as IgG4-related disease (IgG4-RD) as it was shown in the retrospective cohorts from Japan (10 out of 17) [7], USA (13 out of 23) [15], Argentina (10 out of 19) [16], South Korea (9 out of 19) [17] or Denmark (19 out of 42) [18] proved that in general nearly half of the cases or even more than 50% of patients with RPF showed the spectrum of IgG4-RD histopathological findings. In Greek cohort serum IgG4 at diagnosis was evaluated in 36 out of 67 patients with RFP and 36% of them had elevated levels [19]. Perioaortic fibrosis was more common in IgG4-related RPF relative to patients with non IgG4-related RPF in Argentinian and Danish study [16,18]. Kasashima et al. [20] retrospectively compared the clinical, serological, and pathological features of 24 patients with IgG4-related aortic aneurysms (IgG4-AAs), 8 with periaortitis (IgG4-PA), and 10 with retroperitoneal fibrosis (IgG4-RF). They found that clinical symptoms, such as low-grade fever, abdominal/lumber pain, and anemia, IL-6 and C-reactive protein (CRP) were significantly higher in IgG4-AAs and IgG4-PA than in IgG4-RF. In our study, CRP was significantly higher in urorenal subtype, and hemoglobin was not significantly different, despite significantly worse kidney function.

In one of the analysis of 179 patients with IgG4-RD, localization of periaortitis/periaortitis predominantly occurred at the infra-renal artery portion of the abdominal aorta, affected older IgG4-RD onset patients, and was prevalent in highly active disease states [21]. In the biggest prospective study in China with the participation of 587 patients with IgG4-RD, the involvement of large vessels was noted in 15.2% (89 patients), whereas thoracic aorta and other middle-sized artery were inflamed in 13.5% each [22,23]. The result of this study indicates that the spectrum of vascular changes in the course of IgG4-disease is not very common. However, in our study, vascular subtype was more prevalent. It should be stressed that RPF with IgG4 seems to be overlooked and the diagnosis of IgG4-related diseases is also challenging in everyday clinical practice.

 We enrolled in the study all the patients starting from 2005. By this time, the awareness of IgG4-RD was low and immunohistochemistry was not usually performed to rule out this diagnosis. Due to retrospective analysis, IgG4 levels are not available for all patients as it was not a standard assessment of suspected RPF in our departments. Nowadays we introduce assessment of IgG4 levels as a part of diagnostic procedure. Although, the treatment modalities of IgG4-related RPF do not differ much from those of idiopathic RPF, differentiation between the two diseases is essential [24]. The availability of serum IgG4 levels for monitoring treatment response and follow-up can curtail the repeated radiological imaging and associated contrast exposure [25]. Second, the diagnosis of IgG4-related RPF should alert the clinician to look out for extraretroperitoneal diseases on follow-up of this multiorgan disease [26]. Biopsy is to be considered also in settings such as atypical location of the mass (e.g., not peri-aortoiliac but instead in a pelvic, isolated peri-ureteral, or peri-bladder location), suspicion of an underlying malignancy or infection on the basis of clinical and laboratory findings, bulky appearance of the retroperitoneal tissue, extension above the origin of the renal arteries, or anterior displacement of the aorta by CT or MRI (suggestive of possible malignancy), and if local expertise with radiologic diagnosis of RPF is limited (tissue confirmation is needed for diagnosis). However, in our patients due to localization no biopsy was performed.

Obstructive uropathy occurs in ∼30% of IAAA, highlighting the importance of rapid recognition and intervention as shown by Stone and Fankhauser [27]. They presented the experience of 69 IAAA treated by open and endovascular methods with results supporting the use of endovascular aneurysm repair (EVAR) for IAAA. They also stressed that retroperitoneal inflammation may not subside post-operatively in patients IAAA by undergoing EVAR because, they also underlined the rationale for treating the obstructive uropathy and AKI prior to endovascular repair. Firstly, it allowed peri-operative optimization of organ function. Furthermore, due to the severity of AKI, it was felt necessary to treat prior to endovascular repair of the AAA to mitigate the effects of contrast-induced nephropathy. As shown, post-contrast AKI is not a great risk for patients, even those with chronic kidney disease [28]. Azizi et al. [11] found one case of IAAA among 12 cases of RPF. They stressed on the basis of this one case that regression of the fibrous plaque remains controversial in RPF secondary to the aortic aneurysm, even after surgical treatment of the latter. They also underlined that in this study, patient with RPF secondary to aorta aneurysm and whose diameter did not exceed 5 mm did not warrant endovascular treatment, and there was a regression of the plate under corticosteroid therapy itself. Aneurysmal dilation (infrarenal aortic diameter ≥30 mm) was frequently present in 25% of patients in the 53 consecutive patients with a diagnosis of idiopathic RPF at our tertiary care referral center in Netherlands from April 1998 through January 2008 [29].

In our center, cardiology subtype (vascular) was predominant. It may be due to the fact that we have two departments of vascular surgery with great expertise in endovascular aneurysms repair surgery and they have lots of referral all over the country. First of all, presented subtypes reflect logistical and clinical reasons. Many patients with peri-aortic infiltration and hydronephrosis due to ureteral obstruction are referred to nephrological or urological departments. In turn, patients with RPF and dominant aortic aneurysms used to be referred to surgical or cardiology departments. Initial diagnosis and treatment, often including surgery, are carried out in appropriate types of units. Moreover, it is the appropriate specialists who assess patients’ prognosis and conduct their further monitoring. Of course, in many patients, both forms coexist and such patients should be treated collectively. Although the pathogenesis of both forms of RPF is still largely unknown, the division of RPF may result from the clinical course of the disease: inflammatory aneurysm and/or obstructive uropathy. Currently, it is difficult to indicate any additional differences that determine the division. Hence, the presented study is an attempt to assess the potential differences between our patients with two subtypes of the disease. So far, it is not clear why aortic aneurysm is dominant in some patients with RPF, while renal complications predominate in others. Despite, the fact that the first description of RPF is more than 100 year old and came from the case report of Albarran in 1905 [30], there is still no answer in the published literature on the pathogenesis of this rare entity. Mainly urorenal presentation is reported, although some associations between IAAA and RPF were reported [11–13] in small series. Therefore, taking into account our long-term clinical experience we presented two subpopulations with RPF in the recent review [2]. Although glucocorticosteroids have a beneficial effect in most RPF patients, an influence of other drug classes including mycophenolate mofetil, cyclophosphamide, azathioprine, methotrexate, cyclosporine and colchicine is still not established. The effect of these drugs in both subgroups requires further studies.

Due to the diversity of symptoms, diagnosis of RPF becomes a challenge for specialists in internal medicine, nephrology, cardiology, angiology, urology, etc. [31]. Nowadays, we can offer a multidisciplinary team approach with clinical and diagnostic experience in both primary and secondary RPF as well as the two major subtypes. In addition, now we do include IgG-4 measurement to exclude or confirm IgG4-related RPF. Centers specialized in rare diseases in collaboration with other units and a referral system yield the best possible outcomes.

## References

[1] Tanaka T, Masumori N. Current approach to diagnosis and management of retroperitoneal fibrosis. Int J Urol. 2020;27(5):387–394. 32166828 10.1111/iju.14218

[2] Łoń I, Wieliczko M, Lewandowski J, et al. Retroperitoneal fibrosis is still underdiagnosed entity with poor prognosis. Kidney Blood Press Res. 2022;47(3):151–162. Epub ahead of print. 34915518 10.1159/000521423

[3] Dalainas I, Nano G, Ranucci M. Inflammatory abdominal aortic aneurysms. A 20-year experience. J Cardiovasc Surg (Torino). 2007;48:305–308. 17505434

[4] Rasmussen TE, Hallet JW, Jr. Inflammatory aortic aneurysms. A clinical review with new perspectives in pathogenesis. Ann Surg. 1997;225(2):155–164. 9065292 10.1097/00000658-199702000-00003 PMC1190644

[5] Uibu T, Oksa P, Auvinen A, et al. Asbestos exposure as a risk factor for retroperitoneal fibosis. Lancet. 2004;363(9419):1422–1426. 15121404 10.1016/S0140-6736(04)16100-X

[6] Goldoni M, Bonini S, Urban ML, et al. Asbestos and smoking as risk factors for idiopathic retroperitoneal fibrosis: a case-control study. Ann Intern Med. 2014;161(3):181–188. 25089862 10.7326/M13-2648

[7] Zen Y, Onodera M, Inoue D, et al. Retroperitoneal fibrosis: a clinicopathologic study with respect to immunoglobulin G4. Am J Surg Pathol. 2009;33(12):1833–1839. 19950407 10.1097/pas.0b013e3181b72882

[8] Tzou M, Gazeley DJ, Mason PJ. Retroperitoneal fibrosis. Vasc Med. 2014;19(5):407–414. 25161213 10.1177/1358863X14546160

[9] Palmisano A, Maritati F, Vaglio A. Chronic periaortitis: an update. Curr Rheumatol Rep. 2018;20(12):80. 30397845 10.1007/s11926-018-0789-2

[10] Jois RN, Gaffney K, Marshall T, et al. Chronic periaortitis. Rheumatology (Oxford). 2004;43(11):1441–1446. 15266064 10.1093/rheumatology/keh326

[11] Azizi M, Zajjari Y, Rafik H, et al. Retroperitoneal fibrosis in the military hospital of Morocco. Saudi J Kidney Dis Transpl. 2020;31(1):169–175. 32129210 10.4103/1319-2442.279937

[12] Kim Y, Ghaly P, Iliopoulos J, et al. Management of an inflammatory abdominal aortic aneurysm causing ureteric obstruction: a case report. J Surg Case Rep. 2020;2020(11):rjaa457. 33294158 10.1093/jscr/rjaa457 PMC7700772

[13] Blazekovic R, Planinc M, Catic J, et al. Immunoglobulin G4 inflammatory aortic aneurysm mimicking acute aortic syndrome. Ann Thorac Surg. 2019;108(3):e179–e181. 30771330 10.1016/j.athoracsur.2019.01.029

[14] Yardimci GK, Ardali Düzgün S, Farisogullari B, et al. Comprehensive assessment and outcomes of patients with chronic periaortitis. Clin Exp Rheumatol. 2022; Mar 18. Epub ahead of print. DOI:10.55563/clinexprheumatol/araba3 35349413

[15] Khosroshahi A, Carruthers MN, Stone JH, et al. Rethinking Ormond's disease: "idiopathic" retroperitoneal fibrosis in the era of IgG4-related disease. Medicine (Baltimore). 2013;92(2):82–91. 23429355 10.1097/MD.0b013e318289610f PMC4553983

[16] Angarola E, Valeo Chulvi M, Peuchot V, et al. Immunoglobulin G4-related retroperitoneal fibrosis. Medicina (B Aires). 2022;82(1):91–98. 35037866

[17] Koo BS, Koh YW, Hong S, et al. Clinicopathologic characteristics of IgG4-related retroperitoneal fibrosis among patients initially diagnosed as having idiopathic retroperitoneal fibrosis. Mod Rheumatol. 2015;25(2):194–198. 25036229 10.3109/14397595.2014.931908

[18] Lomborg N, Jakobsen M, Bode CS, et al. IgG4-related disease in patients with newly diagnosed idiopathic retroperitoneal fibrosis: a population-based Danish study. Scand J Rheumatol. 2019;48(4):320–325. 30931680 10.1080/03009742.2018.1551963

[19] Zampeli E, Venetsanopoulou AI, Christaki S, et al. Idiopathic retroperitoneal fibrosis: clinical features, treatment modalities, relapse rate in greek patients and a review of the literature. Clin Exp Rheumatol. 2021; Nov 11. Epub ahead of print. DOI:10.55563/clinexprheumatol/umzfau 34796838

[20] Kasashima S, Kawashima A, Kasashima F, et al. Inflammatory features, including symptoms, increased serum interleukin-6, and C-reactive protein, in IgG4-related vascular diseases. Heart Vessels. 2018;33(12):1471–1481. 29931542 10.1007/s00380-018-1203-8

[21] Ozawa M, Fujinaga Y, Asano J, et al. Clinical features of IgG4-related periaortitis/periarteritis based on the analysis of 179 patients with IgG4-related disease: a case-control study. Arthritis Res Ther. 2017;19(1):223. 28978347 10.1186/s13075-017-1432-8 PMC5628426

[22] Peng L, Zhang P, Li J, et al. IgG4-related aortitis/periaortitis and periarteritis: a distinct spectrum of IgG4-related disease. Arthritis Res Ther. 2020;22(1):103. 32366271 10.1186/s13075-020-02197-w PMC7197178

[23] Li J, Peng Y, Zhang Y, et al. Identifying clinical subgroups in IgG4-related disease patients using cluster analysis and IgG4-RD composite score. Arthritis Res Ther. 2020;22(1):7. 31924265 10.1186/s13075-019-2090-9 PMC6954570

[24] Martín-Nares E, Hernández-Molina G, Baenas DF, et al. IgG4-related disease: mimickers and diagnostic pitfalls. J Clin Rheumatol. 2022;28(2):e596–e604. Epub ahead of print. 34538846 10.1097/RHU.0000000000001787

[25] Chandna A, Sharma AP, Pareek T, et al. IgG4-related retroperitoneal fibrosis: an emerging masquerader with a sinister presentation. Urology. 2019;133:16–20. 31229513 10.1016/j.urology.2019.06.007

[26] Huda SA, Kahlown SA, Elder R, et al. Immunoglobulin G-4-related retroperitoneal fibrosis. J Investig Med High Impact Case Rep. 2021; 9:23247096211022487. 10.1177/23247096211022487 PMC8182217 34088232

[27] Stone WM, Fankhauser GT. Inflammatory aneurysms treated with EVAR. Semin Vasc Surg. 2012;25(4):227–231. 23206570 10.1053/j.semvascsurg.2012.09.008

[28] Chomicka I, Kwiatkowska M, Lesniak A, et al. Post-contrast acute kidney injury in patients with various stages of chronic kidney disease-is fear justified? Toxins (Basel). 2021;13(6):395. 34206100 10.3390/toxins13060395 PMC8226462

[29] van Bommel EFH, Jansen I, Hendriksz TR, et al. Idiopathic retroperitoneal fibrosis: prospective evaluation of incidence and clinicoradiologic presentation. Medicine (Baltimore). 2009;88(4):193–201. 19593223 10.1097/MD.0b013e3181afc420

[30] Albarran J. Retention renale par peri-ureterite: liberation externe de l’uretere. Assoc Fr Urol. 1905;9:511–517.

[31] Vaglio A, Maritati F. Idiopathic retroperitoneal fibrosis. J Am Soc Nephrol. 2016;27(7):1880–1889. 26860343 10.1681/ASN.2015101110 PMC4926988

